# Meta-analysis of limb salvage versus amputation for treating high-grade and localized osteosarcoma in patients with pathological fracture

**DOI:** 10.3892/etm.2012.685

**Published:** 2012-08-28

**Authors:** KE YIN, QIANDE LIAO, DA ZHONG, JIE DING, BING NIU, QIUPPING LONG, DENGFENG DING

**Affiliations:** 1 Departments of Orthopaedics; 2 General Surgery, Xiangya Hospital, Central South University, Changsha, Hunan 410008, P.R. China

**Keywords:** osteosarcoma, pathological fracture, limb salvage, amputation, meta-analysis

## Abstract

The goal of this study was to determine outcomes related to limb salvage vs. amputation for treating high-grade and localized osteosarcoma in patients with pathological fractures. Literature search was conducted using Medline, Embase and the Cochrane Database. Two reviewers independently assessed all eligible publications. The primary outcome measurement was pooled odds ratio (OR) and 95% confidence interval (CI) for the risk of local recurrence, 5-year overall survival rate and metastatic occurrence calculated through the fixed-effects method. Seven eligible studies were identified, which included a total of 284 patients. The risk for local recurrence and 5-year overall survival rate did not differ significantly (P>0.05) between the limb salvage group and amputation group, with an OR of 1.48 (95% CI, 0.67–3.30) and 1.85 (95% CI, 0.86–3.98), respectively. The risk for metastatic occurrence differed significantly (P<0.05), with an OR of 0.30 (95% CI, 0.10–0.91). The occurrence of a pathological fracture is not regarded as an absolute contraindication to limb salvage in patients with high-grade and localized osteosarcoma. Limb salvage as an alternative for treating high-grade and localized osteosarcoma in patients with pathological fracture does not greatly increase the risk for local recurrence or 5-year overall survival rate compared to amputation and has a lower risk for metastatic occurrence.

## Introduction

Limb salvage is beneficial for patients with osteosarcoma when complete tumor anatomical resection is possible and neoadjuvant or adjuvant chemotherapy is used, not only for the function of the limb itself, but also for the psychology of the patient (1). The presence of a pathologic fracture in osteosarcoma is often difficult to treat and has been historically associated with a poor outcome (2,3). The incidence of pathological fractures, either at diagnosis or during preoperative treatment, is between 5 and 10% (4–6). Limb salvage has been regarded as an absolute contraindication when a pathological fracture is present for two main reasons (7,8): first, the fracture often causes local hematoma formation, which is conducive for the spreading of tumor cells to adjacent tissues and subsidiary joints; and second, microcirculation damage can promote transfer of the tumor.

It is generally accepted that limb salvage treatment is indicated for primary malignant degree and localized osteosarcoma (such as Enneking stage I osteosarcoma), and surgical amputation is warranted in cases of high malignancy osteosarcoma (such as Enneking stage III osteosarcoma). In addition, most clinicians accept limb salvage treatment for high-grade and localized osteosarcoma (such as Enneking stage II osteosarcoma), but the presence of a pathologic fracture makes the surgical decision difficult. Some surgeons believe that immediate and aggressive removal of the tumor may halt fracture-induced disease progression and that early amputation is a surgical option for all osteosarcoma patients with a pathologic fracture (4,8–10). However, other surgeons believe that limb salvage has recently become an alternative for treating high-grade and localized osteosarcoma with pathological fracture due to the acceptable clinical outcome (7,11–13). However, only a few studies have specifically compared the outcome of limb salvage with that of amputation in osteosarcoma patients with a pathologic fracture (7–14). Moreover, it has not been determined whether limb salvage has a negative influence on survival or local recurrence, since the studies have produced contradictory results.

In the present study, we performed a meta-analysis to determine the local recurrence, 5-year overall survival rate, and metastatic occurrence after limb salvage compared to amputation in order to provide a clear approach for clinicians when choosing a surgical option, especially for high-grade and localized osteosarcoma patients with a pathologic fracture.

## Materials and methods

### Search strategy

We performed a systematic search of Medline, Embase and the Cochrane Database in November, 2011 to identify published studies related to osteosarcoma and fracture. The medical search terms ‘osteosarcoma’, ‘pathologic fracture’, ‘limb salvage’ and ‘amputation’ were combined. No language or other restrictions were placed on the search. Furthermore, references cited in published original and review articles were examined until no further study could be identified. Authors of the retrieved articles were contacted when necessary and were asked to provide additional information.

### Inclusion and exclusion criteria

Articles were included if they reported on studies that included ‘limb salvage’ or ‘amputation’ groups in ‘high-grade and localized osteosarcoma’ in patients with pathological fracture and provided sufficient data to calculate an odds ratio (OR) and corresponding 95% confidence interval (CI). Articles were excluded if the only reported outcome measurements related to ‘limb salvage’ or ‘amputation’ groups without a control group. Case report articles were also excluded due to the small patient numbers. Articles reporting on the same cohort group from the same institution were limited to the most recent publication.

### Quality assessment

Eligible articles were assessed for quality by 2 independent reviewers. The quality of studies in this meta-analysis was assessed using the Newcastle-Ottawa scale quality assessment as recommended by the Cochrane Non-Randomized Studies Methods Working Group. This scale allocates a maximum of 9 points for quality of selection, comparability, exposure, and outcome of study participants. Given the variability in the quality of the observational studies found in our initial literature search, we considered studies that met 5 or more of the Newcastle-Ottawa scale scores criteria as good quality and therefore included only these studies in our meta-analysis.

### Data extraction

Data were extracted from each article by two authors of this study using a structured sheet and then entered into a database. Study characteristics extracted from each manuscript included the country, year of publication, number of cases and controls, study period, age, gender and follow-up. Any disagreement between researchers was resolved by continuing discussions until a consensus was reached.

### Outcome measures

The primary outcome measurement used for analysis was local recurrence. The secondary outcome measurement was the 5-year overall survival rate. In addition, the occurrence of metastasis was assessed as a third outcome measurement.

### Statistical analysis

Pooled OR and 95% CI for dichotomous data were estimated according to the inverse of variance method available through the Review Manager Software (version 5.0 for Windows), which forms the Cochrane Information Management System (IMS). We assessed the heterogeneity of trial results by inspecting graphical presentations and by calculating an I^2^ statistic of inconsistency. We also reported the *Z* statistic for the overall effect. Statistically significant heterogeneity was defined as an I^2^ value >0.05. We used a fixed-effect model to pool the OR, except in the event of statistically significant heterogeneity, in which case a random-effects model was used.

## Results

### Literature search

**Figure 1:**
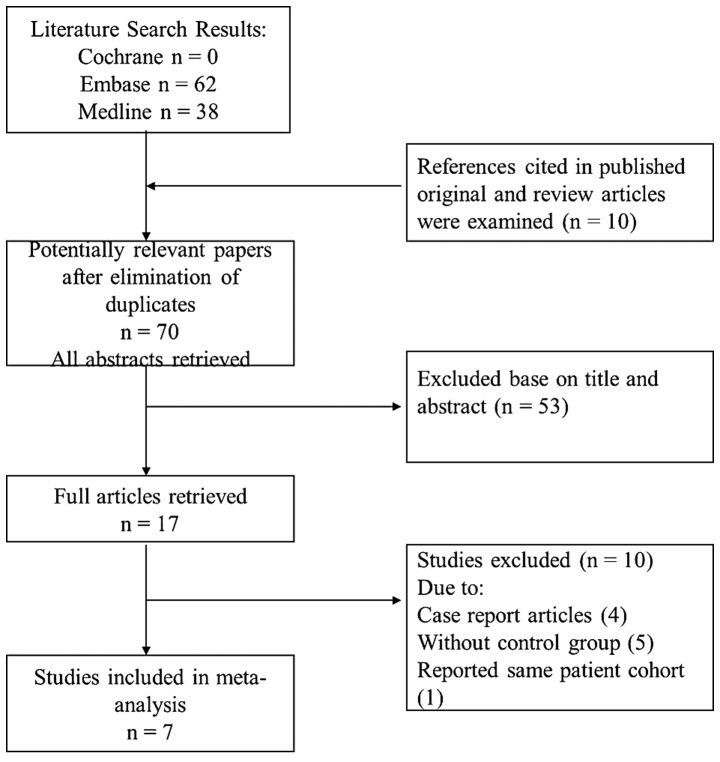
Flow chart of studies retrieved and studies excluded.

We identified 70 potentially relevant articles in the primary literature search (Fig. 1), of which 7 articles (8,15–20) met the inclusion criteria. No randomized controlled trials were identified. Two articles reported on the same patient cohort, and as result the more recent article was included (14,20).

### Study characteristics and quality assessment

All of the 7 studies included were retrospective cohort studies. The publication dates ranged from 1996 to 2010. A total of 284 patients were included in the studies, 200 of whom underwent limb salvage and 84 of whom underwent amputation. The patient demographics, follow-up, and normal characteristics are listed in Table I. There were 5 high-quality studies, as determined by a Newcastle-Ottawa scale score of 6 or higher (71%) (Table II).

### Meta-analysis of local recurrence

**Figure 2:**
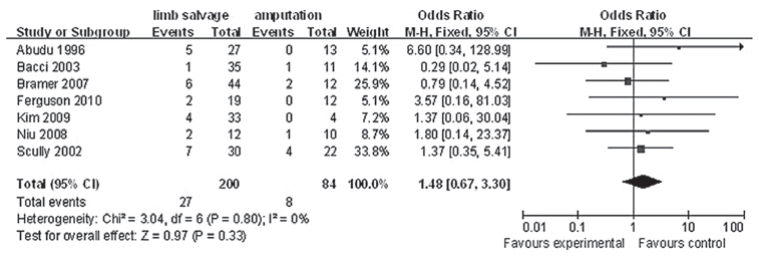
Forest plot of comparison: local recurrence of limb salvage vs. amputation for the treatment of osteosarcoma in patients with pathological fracture.

A total of 284 patients from the 7 trials were classified as having been analyzed for local recurrence. In a meta-analysis of these 7 trials, there were no significant differences (P>0.05) between patients in the limb salvage group and the amputation group (OR, 1.48; 95% CI, 0.67–3.30; *Z*=0.97; P=0.33), and there was no heterogeneity among these trials with respect to overall survival (P=0.80; I^2^=0%) (Fig. 2).

### Meta-analysis of 5-year overall survival

**Figure 3:**
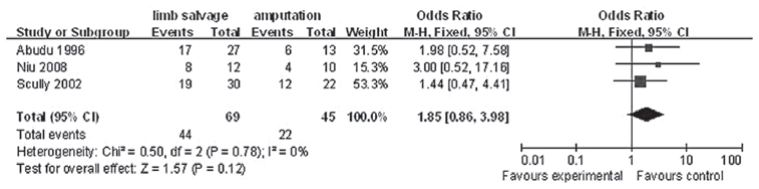
Forest plot of comparison: 5-year overall survival of limb salvage vs. amputation for the treatment of osteosarcoma in patients with pathological fracture.

A total of 114 patients from 3 trials were classified as having been analyzed for 5-year overall survival. The meta-analysis of these patients showed no significant differences (P>0.05) between the limb salvage group and the amputation group (OR, 1.85; 95% CI, 0.86–3.98; *Z*=1.57; P=0.12), and there was no heterogeneity among these trials with respect to overall survival (P=0.78; *I^2^*=0%) (Fig. 3).

### Meta-analysis of metastatic occurrence

**Figure 4:**
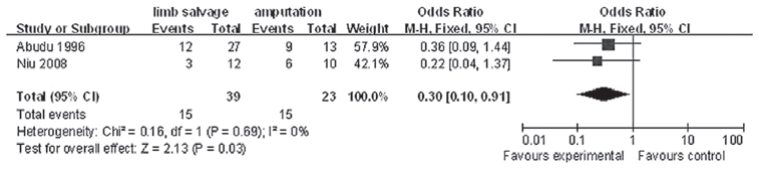
Forest plot of comparison: metastatic occurrence in patients receiving limb salvage vs. amputation for the treatment of osteosarcoma in patients with pathological fracture.

A total of 62 patients from 3 trials were classified as having been analyzed for metastatic occurrence. The meta-analysis of these patients showed significant differences (P<0.05) between the limb salvage group and the amputation group (OR, 0.30; 95% CI, 0.10–0.91; *Z*=2.13; P=0.03), and there was no heterogeneity among these trials with respect to overall survival (P=0.69; I^2^=0%) (Fig. 4).

### Study heterogeneity and publication bias

**Figure 5:**
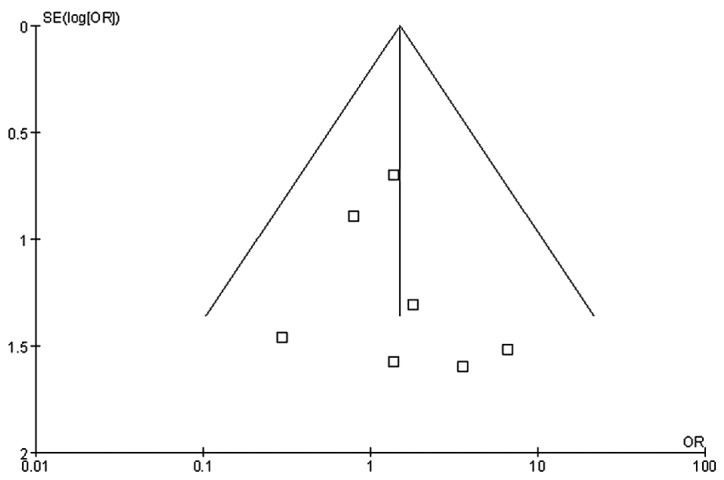
Funnel plot of comparison: local recurrence of limb salvage vs. amputation on treating osteosarcoma in patients with pathological fracture.

The P-value for heterogeneity was 3.04 and the variability (I^2^) in results across all studies as a result of the true differences in treatment effect was 0%, which indicated no heterogeneity. In addition, the funnel plot for all studies was symmetrical (Fig. 5), indicating that the results of all of the studies were expected, as all studies fell evenly within the top of the inverted funnel. This funnel plot pattern also indicated that there was no publication bias.

## Discussion

The benefit of limb salvage treatment for osteosarcoma is clear, not only for primary malignancy and localized osteosarcoma cases, but also for high-grade and localized osteosarcoma cases, since previous studies have shown promising results (7,11,21,22). However, when a pathological fracture is present in cases of high-grade and localized osteosarcoma, the choice of limb salvage over amputation becomes more complicated. First, does the presence of a pathological fracture make limb salvage treatment too complicated and risky to attempt? Second, are the rates of local recurrence and metastasis higher after limb salvage compared to amputation? Third, do these patients have a lower survival rate after limb salvage compared to amputation?

The meta-analysis in this study found no significant difference in local recurrence (OR, 1.48; 95% CI, 0.67–3.30; P=0.33) between limb salvage and amputation methods for treating osteosarcoma with pathologic fracture. Niu *et al* (19) found local recurrence rates of 16 and 10% in a group of 22 patients with osteosarcoma in the extremities and pathologic fracture who received limb salvage or amputation, respectively. The authors concluded that pathologic fractures can be safely managed by limb salvage treatment with an acceptable rate of local recurrence. Another study reported only two recurrences in a group of 46 patients, with one occurring after limb salvage and one after amputation (15). In contrast to these studies, Scully *et al* (14) reviewed 18 patients with pathologic fractures in osteosarcoma and found that the local recurrence rate in patients undergoing limb salvage surgery was markedly higher than in patients undergoing amputation.

The meta-analysis in this study found no significant difference in 5-year overall survival (OR, 1.85; 95% CI, 0.86–3.98; P=0.12) between limb salvage and amputation methods for treating osteosarcoma patients with a pathologic fracture. Abudu *et al* (8) reported that the amputation provided better eradication of local tumor than limb salvage in a group of 40 patients with localized osteosarcoma presenting with pathologic fracture who had been treated with neo-adjuvant chemotherapy and surgery; however, the authors found that amputation did not prolong the 5-year overall survival. In addition, a retrospective analysis of approximately 30 osteosarcoma patients with pathological fracture out of 336 treated patients found that limb salvage treatment did not affect the survival rate (23).

The meta-analysis in this study found a significant difference in metastatic occurrence (OR, 0.30; 95% CI, 0.10–0.91; P=0.03) between limb salvage and amputation methods for treating osteosarcoma patients with a pathological fracture. Niu *et al* (19) found metastasis occurred in 25% (3/12) and 60% (6/10) of cases in a group of 22 patients with osteosarcoma in the extremities and pathological fracture who received limb salvage or amputation, respectively. Abudu *et al* (8) reported that treatment by limb salvage or amputation did not significantly influence the development of metastases, which were noted in 12 of the 27 patients with limb salvage and 9 of the 13 who had amputation. Although the meta-analysis showed a significant difference, only two studies can be incorporated, which included 66 patients. Therefore, it is possible that objectivity could be lost due to the low number of incorporated studies or patients analyzed. Another possible reason is that the occurrence of metastases in patients receiving limb salvage treatment was markedly lower than that in patients receiving an amputation. Thus, additional high-quality, randomized controlled studies are needed to confirm these findings.

It has been previously shown that factors other than the surgical choice of limb salvage or amputation can have an impact on patient outcome in these cases, including tumor size (24), poor response to chemotherapy (25), serum lactate dehydrogenase levels (26,27), anatomical location (9), unstable fracture without healing, age (28), histologic subtype (26), and the timing of the fracture. For high-grade and localized osteosarcoma, the occurrence of a pathological fracture is not regarded as an absolute contraindication to limb salvage.

### Limitations and strengths

Some limitations existed for this meta-analysis that were inherent to the nature of the available data. First, we were only able to include a few studies in the analysis. In addition, all of the included studies had retrospective designs with small sample sizes that were subject to systematic and random biases. With respect to possible selection bias, the included studies had patients with poor response to chemotherapy and larger tumors who were more likely to undergo amputation. This was likely due to the tendency to protect limb function in patients with smaller tumors and the concern for local recurrence in patients treated with chemotherapy. However, verification of this selection bias was not possible in a retrospective manner, and therefore these biases were not found across all studies. The small sample sizes and the small number of included studies is more likely the reason for the failure to detect heterogeneity if it did exist, since the test for heterogeneity is powered low in this type of situation. In addition, the number of events for both primary outcomes was very low. Therefore, the findings of this meta-analysis should be interpreted with caution despite representing the best available evidence to date.

In summary, based on the findings of our meta-analysis, we conclude that limb salvage treatment can be safely used in high-grade and localized osteosarcoma patients with pathological fracture without increasing the risk for local recurrence or decreasing the 5-year overall survival rate. In addition, the development of metastases may be lower in patients receiving limb salvage treatment compared to patients receiving an amputation. Importantly, the occurrence of a pathological fracture is not regarded as an absolute contraindication to limb salvage in patients with high-grade and localized osteosarcoma. Therefore, in the absence of randomized data, this meta-analysis provides the best available evidence to support the use of limb salvage as a surgical alternative for treating osteosarcoma patients with pathological fracture.

## Figures and Tables

**Table I:** Characteristics of the seven included studies. Author/(Ref.)

Author/(Ref.)	Abudu *et al* (8)	Bacci *et al* (15)	Bramer *et al* (16)	Ferguson *et al* (17)	Kim *et al* (18)	Niu *et al* (19)	Scully *et al* (20)
Country	England	Italy	UK	Canada	Korea	China	USA
No. of patients	40	46	56	31	37	22	52
Limb salvage, n (%)	27 (68)	35 (76)	44 (79)	19 (61)	33 (89)	12 (55)	30 (58)
Amputation, n (%)	13 (32)	11 (24)	12 (21)	12 (39)	4 (11)	10 (45)	22 (52)
Study period	1975–1994	1983–1999	1983–2003	1989–2006	-	1992–2001	1977–1996
Male, n (%)	26 (65)	24 (52)	36 (64)	14 (45)	26 (70)	15 (68)	28 (54)
Female, n (%)	14 (35)	22 (48)	20 (36)	17 (55)	11 (30)	7 (32)	24 (46)
Median age (range), years	18 (2–46)	11 (3–20)	16 (4–57)	30 (11-8)	-	18 (3–36)	23.5±17.4^a^
Enneking stage	Stage-IIB	Stage-IIB	-	-	Stage-II	Stage-IIB	Stage-IIB
Follow-up (range), months	55 (8–175)	132 (36–240)	117 (7–252)	-	43 (10–228)	54.7 (8–146)	54 (6-152.4)
Proximal tumor, n (%)	18 (45)	21 (45)	23 (41)	19 (61)	20 (54)	12 (55)	25 (48)
Adequate margin, n (%)	7 (18)	6 (13)	35 (63)	-	8 (22)	-	2 (4)
Poor chemotherapy response^b^, n (%)	-	12 (26)	43 (78)	-	23 (62)	-	29 (64)
Displaced fracture, n (%)	-	8 (17)	-	22 (71)	9 (24)	4 (18)	16 (31)
Local recurrence, n (%)							
Limb salvage	5 (19)	1 (3)	6 (14)	2 (6)	4 (11)	2 (9)	7 (23)
Amputation	0 (0)	1 (9)	2 (17)	0 (0)	0 (0)	1 (10)	4 (18)
5-year survival, n (%)							
Limb salvage	17 (63)	-	-	-	-	8 (66)	19 (63)
Amputation	6 (46)	-	-	-	-	4 (40)	12 (55)
Metastatic occurence, n (%)							
Limb salvage	12 (44)	-	-	-	-	3 (25)	-
Amputation	9 (69)	-	-	-	-	6 (60)	-

a The values are provided as the mean and standard deviation.

b Defined as the percentage of tumor necrosis <90%.

**Table II:** Quality assessment for the seven included studies using the Newcastle-Ottawa quality assessment scale.

	Selection			Comparability			Exposure			
Author/(Refs.)	1	2	3	4	5	6	7	8	9	NOS
Abudu *et al* (8)	*	-	*	*	*	*	*	*	*	8*
Bacci *et al* (15)	*	-	*	*	*	*	*	-	-	6*
Bramer *et al* (16)	*	*	*	*	-	*	*	-	-	6*
Ferguson *et al* (17)	*	-	*	*	-	*	*	-	-	5*
Kim *et al* (18)	*	-	*	*	-	*	*	-	-	5*
Niu *et al* (19)	*	-	*	*	*	*	*	*	*	8*
Scully *et al* (20)	*	*	*	*	*	*	*	*	-	8*

1, inclusion criteria;

2, sample size >50;

3, endpoint;

4, anatomical location;

5, Enneking stage;

6, chemotherapy;

7, local recurrence;

8, 5-year overall survival;

9, metastatic;

NOS, Newcastle-Ottawa scale score.
